# Unsuspectingly Vulnerable: A Rapid Evidence Assessment on the vulnerability of University Students to Criminal Exploitation

**DOI:** 10.1177/0306624X251329944

**Published:** 2025-03-25

**Authors:** Isobel Meredith, Joseph L. Davies, Daniel Stubbings, Libby Payne

**Affiliations:** 1 Cardiff Metropolitan University, UK

**Keywords:** university students, criminal exploitation, county lines, organized crime groups, modern slavery

## Abstract

Criminal exploitation is the deliberate coercion of another person into criminal activity for the perpetrator’s advantage. Under the Modern Slavery Act, the umbrella term of criminal exploitation encompasses the County Lines model of drug distribution which can also include perpetrators forcing victims to commit theft, money laundering or to hold weapons. There is limited understanding of how criminal exploitation affects university students. This research therefore aimed to identify the tactics used by Organized Crime Groups to target them, and factors that are shown to increase their vulnerability to exploitation. Five published studies were included in our review which outline a number of tactics and risk factors for criminal exploitation in university students. Our review highlights the scarcity of literature pertaining to prevalence of criminal exploitation in university students and the need for further empirical research.

## Introduction

The United Kingdom (UK) Modern Slavery Act was introduced in 2015 to provide a legal defense for victims of slavery and trafficking and to ensure suitable punishment for perpetrators (UK Parliament, 2015). Within the Act, Section 1 refers to offences of slavery, servitude and forced and compulsory labor, in which offences related to criminal exploitation fall (Crown Prosecution Service, 2023; Walsh, 2023). It is important to recognize the terminology “criminal exploitation” is often used interchangeably with “County Lines” in the literature (Olver & Cockbain, 2021; Centre for Social Justice, 2022). County Lines is a term used to describe the expansion of city-based drug supply to rural areas through “deal lines”; in the Serious Violence Strategy it is outlined that County Line gangs “are likely to exploit children and vulnerable adults to move [and store] the drugs and money and they will often use coercion intimidation, violence (including sexual violence) and weapons” (HM Government, 2018, p. 48). However, the Centre for Social Justice (2022) highlights that criminal exploitation extends beyond County Line offences. They give examples of perpetrators coercing victims to commit acquisitive offences such as burglary, shoplifting and money laundering, or violent offences, including the holding and carrying of weapons.

Historically, research has tended to focus on the criminal exploitation of those under 18 years. In interviews with professionals involved in the response to County Lines exploitation, Olver and Cockbain (2021) noted that despite framing questions inclusively, participants tended to respond in relation to child exploitation, rather than the victimization of adults. It was suggested that this may reflect the academic and practical focus on child exploitation, or the participants’ perception of where the harm is the most severe. Barlow et al. (2022) argued that children are more often prey to exploitation due to the imbalances of power between them and the perpetrator, whereby the needs and aspirations are identified by the perpetrator which are subsequently used to exploit. The authors suggest that this is particularly relevant to children living in poverty where there is increased hardship, higher levels of need, and who may therefore desire protection or financial gain. In fact, the UK Parliament’s inquiry into youth violence estimated 1 in 25 children in the country are currently at risk of criminal exploitation (UK Parliament, 2023). Involvement in criminal activity is reported to have a significantly negative impact on children, including disengagement from education, family breakdowns, and becoming more at risk of experiencing serious violence (Krohn et al., 2011).

Notwithstanding the importance of safeguarding children from criminal exploitation, there also appears to be rising concern in the public media surrounding the exploitation of university students (Barclays, 2023; Hall & Kahn, 2022; Marsh, 2020). Organized Crime Groups (OCGs) are now understood to recruit “clean skins,” that is those without a history with statutory agencies, to decrease detection (O’Hagan & Edmunson, 2021). As such, university students with no criminal record may be able to fly under police radar. University students could also arguably fit the profile of an individual vulnerable to criminal exploitation due to the presence of known risk factors in this population, namely substance abuse, social isolation, mental health vulnerabilities, and financial insecurity.

### Substance Abuse

The National Crime Agency (2017) reported that people who use substances may be at particular risk for criminal exploitation, for example, 61% of police forces reported that people who use drugs were most often exploited by County Line gangs. Approximately 17% of university students reported using illegal substances (Students Organising for Sustainability, 2023) which is double that of the general population (Office for National Statistics, 2023). Data from the National Survey on Drug Use 1975 to 2021 (Patrick et al., 2022) as highlighted that young adults attending university have a higher prevalence of illicit substance use for some substances, compared to their non-university counter parts, such as cocaine, ketamine, opiates (i.e., vicodine), and prescription stimulants without a doctors prescription (i.e., adderall and ritalin). In the case of the latter, it is plausible that the use of stimulants in university students is greater given the association with these substances as study drugs (Sussman et al., 2006). The greater prevalence of these illicit substances could also be explained through the rational choice assumption that students have more opportunities to use illegal substances due to living away from caregivers, often for the first time, and more regular attendance at places associated with substance use, such as pubs and clubs (T. H. Bennett & Holloway, 2015).

Within this context, university students would appear to be suitable targets for OCGs looking to expand their customer base. On the other hand, T. H. Bennett and Holloway (2019) reported that of nearly 8,000 students interviewed, one third of those who had used substances admitted to also selling substances. This was explored by Moyle and Coomber (2019) who reported that universities are a prevalent risk environment for students to transition from substance use into the social supply of substances. In interviews with students who had engaged in supplying drugs to their peers, many reported initially purchasing drugs for a group of friends and distributing them. Once identified as individuals with access to substances, they noted that this often led them to become the primary point of contact, eventually resulting in a more regular involvement in drug supply. On that basis it is plausible that drug distribution by university students may be more linked to independent social supply rather than organized crime involvement.

### Social Isolation and Mental Health

Social isolation and loneliness are also notably prevalent amongst university students; problems with generating and maintaining social connections are one of the most reported stressors for this population (Hurst et al., 2013). The COVID-19 pandemic may have exacerbated this further when students had less opportunity to create social bonds in often unfamiliar environments (Dedryver & Knai, 2021). Unsurprising, social isolation plays a significant role in mental health concerns for students (Thorley, 2017). This is suggested as the result of loneliness and the associated exacerbation of symptoms of anxiety and depression in this population (Richardson et al., 2017). This is concerning as mental health issues are also indicated as predisposing individuals to criminal exploitation, for example, the National Crime Agency (2017) stated that 37% of police regions reported criminal exploitation of adults with mental health concerns. Focusing again on university students, Bantjes et al. (2023) noted that mental health concerns are highly prevalent amongst this group, with it being considered a national priority in the higher education sector. As such, the interconnected risk factors of poor mental health and social isolation may leave university students particularly vulnerable to criminal exploitation (Pearson & Cavener, 2024).

Increased social isolation and the associated impact on mental wellbeing is particularly problematic when considering criminal exploitation risk because criminal groups use grooming tactics to exploit victims who lack social connections. Pearson and Cavener (2024) described a process of exploitation where the victim is introduced to the perpetrator’s “friendship” group and gain a sense of belonging. Once trust is established, the victim is “tested” when they are encouraged to engage in criminal activity. They are then at risk of blackmail and threats, making them feel trapped into the criminal operations. It has been argued that, for some young people, the promise of social capital (i.e., positive connections and bonding between people) may be a more significant motivator for engaging in criminal activity rather than financial benefits, particularly for those with experiences of social or educational exclusion (Joseph, 2017). Whilst for some the promise of social capital may be a significant allure, the influence of financial gain cannot be overstated.

### Financial Insecurity

The promise of financial renumeration is another risk factor associated with vulnerability to criminal exploitation in the general public (Hayman et al., 2024). Within a university student population, this risk factor is particularly pertinent given the significant financial distress incurred consequent to increased student loan costs and the general cost-of-living crisis (J. Bennett et al., 2023). In fact, 50% of students surveyed in the Student Cost of Living Insight Study reported having financial difficulties, with a quarter taking on new debt to support their living costs (Office for National Statistics, 2022). Furthermore, financial gains made from involvement in criminal activity can give the impression of success, power, and wealth (Fraser et al., 2018). This is significant as Moore et al. (2021) reported that students felt ostracized by social comparison to wealthier peers and wanted to construct their identity around appearing equally privileged. This may be exacerbated by perpetrators glamorizing their lifestyle of “easy” money on social media (Irwin-Rogers, 2019), with university students’ life satisfaction being particularly influenced by comparison to those online (Hawi & Samaha, 2017). It is thus argued that university students are susceptible to victimization due to desire for financial gain, either to ease financial distress or to build social capital with their peers.

Their ignorance of a common tactic used by criminals to launder money might also be increasing their vulnerability. The National Crime Agency (2024) have highlighted that students may be particularly vulnerable to “money muling” (i.e., holding or moving money in exchange for payment or other benefits) or money laundering. A common tactic involves recruiting the “mules” through social media accounts or friends and family members and persuading them to share details of their student bank account to move money.

There is also evidence to suggest that UK universities are aware of this issue and have tried to target harden students against it. For example, the University of Birmingham’s Law School collaborated with a local law firm to raise awareness of money muling, having found that over 52% of students lacked knowledge about what a money mule is (Tenet Law, 2022). Similarly, in 2024, Liverpool John Moores University gave warnings to students about money mule scams during a student finance event. However, this information comes from grey literature sources and does not appear to have been researched robustly.

Financial instability interacts with other risk factors involved in victimization. For example, the National Union of Students (2022) Cost of Living Survey stated that 92% of students surveyed reported negative impacts on their mental health because of the cost-of-living crisis. Additionally, in their study including university students in the United Kingdom, El Ansari et al. (2015) found that feeling financial burden was a significant predictor of occasional use of illicit substances. However, caution should be taken when interpreting these findings given that the authors did not capture the nature of this illicit drug use (i.e., whether the use was misuse or abuse). In any case, it is possible that the interplay between these vulnerability factors could further increase the risk for university students to be criminally exploited.

Given the high prevalence of risk factors in student populations, it is important to consider how they may be exploited. Typical tactics utilized by OCGs can include debt bondage, which involves exploiters providing free goods such as drugs, accommodation, or clothes, and later forcing victims to repay these debts through criminal activity following intimidation, threats, or blackmail (Stone, 2018). This may be an especially fruitful tactic with university students, as material goods may provide them the desired financial and social capital (Joseph, 2017). Provision of drugs may also be particularly tempting for students given their more frequent usage of some substances compared to peers that do not attend university (Patrick et al., 2022). This has led to concern that victims may have their student accommodation “cuckooed” (Hall & Kahn, 2022; Marsh, 2020), described by Jaensch and South (2018) as gang members using the house of the victim as a base for storing and distributing drugs. There has also been consideration of how exploiters coerce victims to commit acquisitive crimes, such as money laundering. It has been reported that perpetrators may post fake job adverts on recruitment or social media sites, promoting “get rich quick” opportunities (Barclays, 2023), attractive for university students looking for flexible work alongside studying. Despite concern, there is limited clarity within the academic literature about the tactics used by OCGs on university students, providing the starting point for this review

### The Current Study

On a national level, it is estimated that criminal exploitation, such as County Lines, by Serious and Organized Crime Groups costs the UK approximately £47 billion annually, threatening national security, disrupting communities, and causing serious personal and financial harm to those victimized (HM Government, 2023). On an individual level, criminal exploitation of students could result in them dropping out of or being expelled from university, gaining a criminal record or being jailed and, in worst cases, might place students at risk of severe violence or death (Marshall, 2024). It is thus vital to understand the nature of criminal exploitation of university students to prevent escalation of this problem. As there is no investigation synthesizing research on criminal exploitation in this population, a Rapid Evidence Assessment (REA) was conducted to explore the following questions:

RQ1: How prevalent is the criminal exploitation of university students?
RQ2: What tactics do OCGs use to exploit university students?
RQ3: What are the characteristics of university students that make them a greater risk to criminal exploitation?

## Methodology

### Design

**Figure 1.**
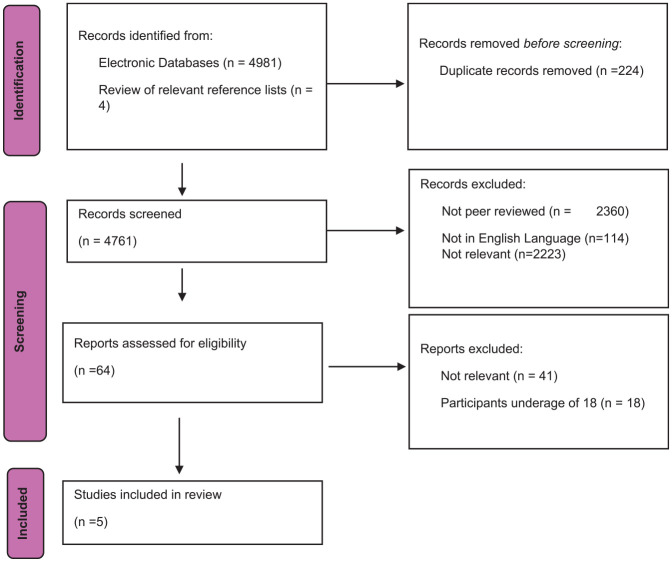
A PRISMA flow chart (Page et al., 2021) demonstrating the screening process.

The research adopted a question-led Rapid Evidence Assessment (REA) methodology. This aims to provide the most comprehensive search of the literature to synthesize the current evidence base (Davies, 2003). Varker et al. (2015) reported that REAs are beneficial as they can objectively comment on the quantity and quality of current publications on a specific topic when bound by resource and time constraints. The review was conducted in accordance with Preferred Reporting Items for Systematic Review and Meta-Analyses (PRISMA) guidance (Page et al., 2021). This was used due to its ability to ensure a transparent, comprehensive, and replicable process for identifying, selecting, and synthesizing the relevant literature. The review follows the PRISMA flow diagram (see Figure 1) and the PRISMA checklist guided the reporting of the methods, results, and discussion.

### Procedure

#### Inclusion and Exclusion Criteria

The search aimed to identify research that explored the criminal exploitation of university students. The criteria for the inclusion of material were as follows:

- Studies published between January 2014 and July 2024
- Studies published in the English Language
- All research methods, including both qualitative and quantitative studies
- Peer Reviewed Studies
- Studies that focused on those under the age of 18 years were excluded from the search, as university students in the U.K. are typically above the age of 18 years.

#### Search Strategy

The current review utilized the Sample, Phenomenon of Interest, Design, Evaluation, Research type (SPIDER) tool (Cooke et al., 2012) to identify the main components of our review and to standardize the search strategy. The search strings combined terms for university students and key forms of criminal exploitation as outlined by the UK Home Office (2023; see Table 1). The literature was identified through systematic searches on ProQuest Central, providing access to 18 databases across multiple subject areas such as the social sciences, education and health, and the Ovid platform to search the PsycINFO database, specializing in research from the behavioral and social sciences. Reference lists of relevant literature were also reviewed to identify research that was not captured within the searches.

**Table 1.** Search Terms Based on the Sample and Phenomenon of Interest Aspects of the SPIDER Tool.

Sample	Phenomenon of interest
Student OR university OR college OR undergraduates OR graduates OR “clean skins” OR higher education	“county line” OR crim* OR “organised crime” OR gang OR traffick* OR cuckoo* OR “trap houses” OR “debt bondage” OR “modern slavery” OR “money laundering” OR “drug running” OR “money mules”

#### Data Abstraction

The screening process for the current study is illustrated in Figure 1. A total of 4,985 references were identified through database searches and reviewing reference lists. During initial screening, 224 duplicate records were removed by automation on the database. The titles were then reviewed in line with inclusion criteria; 2360 were removed as they were not peer reviewed, 114 as they were not in the English language and 2,223 were deemed not relevant to the topic of criminal exploitation of university students. The remaining 64 abstracts or full articles were reviewed; a further 41 were excluded as they were not totally relevant to the subject matter, and 18 were excluded as they included participants below the age of 18 years. This left five studies to be included in the review.

#### Assessment of Research Quality

The REA included studies with varying research design yet there is no one tool that will capture this diversity when attempting to consistently assess methodological quality. As such, the Quality Assessment Tool for Studies with Diverse Designs (QATSDD; Sirriyeh et al., 2012) was utilized for quantitative studies included in the review. The QATSDD consists of 16 items, 14 of which can be used in reference to quantitative studies. Each item is scored on a 4-point scale and the score of methodological quality is calculated as a percentage, with higher percentages reflecting a better-quality paper. However, this tool was not appropriate for literature reviews included within the REA. Consequently, the Scale for Assessment of Narrative Review Articles (SANRA; Baethge et al., 2019) was adopted for papers with literature review designs. This is a six-item scale, scored from 0 to 2, with higher scores indicating higher quality. Two members of the research team independently reviewed the quality of studies. Discussions between the two researchers (Initials omitted for blinded review) were held following this and final scores were calculated, with little to no disagreements between researchers. For consistency, within Table 2 percentages have been given to demonstrate the quality of the included research, with clarification of which tool was utilized to assess quality.

**Table 2.** Study Description and Quality of Evidence Assessments.

Source title	Authors	Aim	Design and methods	Quality of evidence	Findings
Flying Under the Radar: How Susceptible Are University Students to County Lines Victimization?	Burt et al. (2022)	To investigate how likely U.K. university students are to participate in county lines scenarios and what makes students vulnerable to this form of exploitation.	Hypothetical scenario vignettes were given to 118 student participants, 5 of which depicted tactics employed by County Line offenders and one control vignette. Participants were asked to rate how likely they would be to engage in the scenario on a 0 to 100 scale. Scores <50 were interpreted as unlikely to engage, and >50 as likely to engage.	69%^a^	• 62% of students reported they would engage in the County Line vignettes compared to only 3% in the control vignette. • Age appeared to be protective as older students demonstrated lower engagement rates in all but one of the County Lines scenarios. • International students may have increased vulnerability to money laundering schemes as they had higher endorsement rates of “a fake job advert” vignette. • Risk factors such as mental health/wellbeing, social isolation, financial distress, substance use, and materialism were also considered. Most showed weak correlations. • The strongest correlation reported was that participants with higher levels of substance abuse were more likely to endorse a “free drugs” scenario.
Drug abuse and trafficking in universities: An emerging social phenomenon.	Nunes et al. (2017)	To explore the phenomena of drug trafficking in Portuguese universities	Literature Review	41.7%^b^	• Students are major consumers of illegal substances and thus drug traffickers will target universities for distribution. • A large proportion of those who sell drugs are also consumers. • Males are more likely to be victim to drug trafficking but there is growing involvement of female students
Money mule recruitment among university students in Malaysia: Awareness perspective.	Vedamanikam and Chethiyar (2020)	To review existing research on recruitment of students in Malaysia as money mules and what factors influence job acceptance	Literature Review and a statistical analysis on cases recorded by law enforcement	58.3%^b^	• Risk factors for students becoming money mules include heavy internet or social media usage, desire for financial gain/flexible work and reduced awareness of job criteria. • Perpetrators create fake online job advertisements of roles to lure students into becoming money mules. • They also cultivate “relationships” with university students online to convince them to transfer funds or open bank accounts.
Job acceptance in money mule recruitment: theoretical view on the rewards.	Vedamanikam et al. (2022)	To explore how rewards influence money mule job acceptance in students from Malaysia	Literature review	58.3%^b^	• Students are targeted by perpetrators as they are attracted to “easy” financial reward and have limited ability to decode criminal intentions • Students can fall victim to fake job advertisements if they offer flexibility and financial rewards.
County Lines and Criminal Exploitation of UK University Students.	Hall et al. (2022)	To explore the prevalence of county lines exploitation of U.K. students and students’ experience of drug use in the context of County Lines	Freedom of Information Requests were sent to 127 public universities in the U.K; 7 Universities held information on County Lines. 140 student participants were given a questionnaire focused on drug use and supply, social media usage, and accessing of support when concerned about own or another’s drug use	40.5%^a^	• Between the years 2017 to 2021, there were 42 to 109 students involved in County Lines. • Indicators of debt bondage were noted to be present. • The majority of participants had been exposed to job adverts promoting “get money quick” or money mule schemes. • Perpetrators use of social media was also considered, noting that 60.7% participants had seen illegal drugs being advertised on sites such as Snapchat. • It was suggested that participants who lived in student accommodation are particularly vulnerable to exploitation, such as having their accommodation “cuckooed.”

a Research quality assessed using QATSDD (Sirriyeh et al., 2012).

b Research quality assessed using SANRA (Baethge et al., 2019).

### Ethics

The REA received ethical approval from Cardiff Metropolitan University School of Sport and Health Sciences (Ref: STA-9633). Despite the REA posing limited ethical concerns due to its use of already published literature, focus was placed on the ethical storage of data in line with General Data Protection Regulation (GDPR, 2016) legislation.

## Results

A summary of studies included in this review, as well as percentage scores of the study quality, can be found in Table 2. Of the five studies included in this review, one was an empirical study using university students in the UK (Burt et al., 2022), another was a technical report that summarized data captured from a number of UK universities (Hall et al., 2022), and three were literature reviews, two of which were from Malaysia (Vedamanikam & Chethiyar, 2020; Vedamanikam et al., 2022), and one from Portugal (Nunes et al., 2017).

### Prevalence of Criminal Exploitation of University Students

Of the five studies included in this review, one included data on the prevalence of involvement in criminal activity, specifically County Lines. In their technical report highlighting the prevalence of County Lines engagement in university students, Hall et al. (2022) found that across seven universities there were a reported 11 to 41 university students who were victims, and 16 to 52 university students who were perpetrators of County Lines exploitation between 2017 and 2021. The authors reported that one institution was not able to provide information on victim-perpetrator details but, including their data, overall County Lines involvement across the six institutions stood between 43 and 109 university students. Whilst it does not provide details on prevalence rates of university students involvement in criminal exploitation, a study by Burt et al. (2022) explored victimization, again specific to County Lines, in 116 university students using hypothetical vignettes. Analysis of responses suggested that 62% of participants were willing to engage in County Lines scenarios, compared to 3% for the control scenario. The authors of this study also collected data on university student demographics and characteristics, with findings highlighting particularly factors predictive of County Lines engagement risk.

### Exploitation Tactics Used by Organized Crime Groups

Three out of the five studies in the current review provided findings on the tactics used by OCGs to exploit university students. Vedamanikam and Chethiyar (2020) for example found in their review that perpetrators would create fake job advertisements online for financially lucrative jobs to lure students into becoming money mules. The authors also reported that perpetrators would cultivate relationships with university students in order to exploit them for money. In a further literature review on fake job advertisements and money muling in the Malaysian context, Vedamanikam et al. (2022) found that students were targeted because they are attracted to the easy financial reward associated with the job, as well as their limited ability to anticipate criminal intentions, such as a lack of awareness of the consequences associated with money laundering.

Similar findings were reported in the UK context, whereby Hall et al. (2022) suggest that the majority of university students that were subject to County Lines exploitation were exposed to job advertisements that promised quick financial reward but that were inadvertently money mule schemes. Hall et al. (2022) also reported the presence of debt bondage, whereby the perpetrator would supply university students with drugs, alcohol, and accommodation at no cost, but would later be told they must repay these costs. Other tactics from the authors report include the use of social media as a means of advertisement, whereby 61% of participants had seen illegal drugs being advertised on popular social media sites.

### Risk Factors for Criminal Exploitation

Four of the five studies included in the current review provided findings on characteristics that present as potential risk factors for criminal exploitation. In their study, Burt et al. (2022) collected details on participants age, gender, ethnicity, level of study, type of student (i.e., UK, European, international), location (i.e., England or Wales), and living situation. Other participant data were also collected such as levels of drugs abuse, mental health well-being, social isolation, financial distress/instability, and scores of materialism. The authors classified of how likely participants were to endorse County Lines scenarios. They found that younger students, students who were male, those who identified as Black/Black British and Asian/Asian British, European and international students, and students living in university halls of residence, sharing accommodation, and “sofa-surfing” were at a greater risk of County Lines involvement. Furthermore, the authors found significant correlations between levels of drug abuse, mental health well-being, materialism, and financial distress and engagement in County Lines scenarios.

Some of the above findings have also been reported in a review of literature pertaining to drug abuse and trafficking in universities from a Portuguese perspective (Nunes et al., 2017). Nunes et al. (2017) report that those involved in drug trafficking were also engaging in drug use and were more likely to be male. Furthermore, in support of Burt et al.’s (2022) findings regarding students living situation, Hall et al. (2022) found that individuals who lived in student accommodation were particularly vulnerable to exploitation, particularly with regards to cuckooing.

Vedamanikam and Chethiyar (2020) offer insight into risk factors from a Malaysian perspective. In their review of literature pertaining to money mule recruitment among students in Malaysia, they reported that an increased risk of becoming a money mule is associated with heavy internet or social media usage, desire for financial gain and flexible work, and reduced awareness of hidden elements of job criteria for jobs that involve money laundering. The authors also noted several methods which OCGs used to exploit university students for money laundering.

## Discussion

The aim of the current REA was to synthesize literature pertaining to criminal exploitation in university students, particularly in relation to prevalence rates, characteristics associated with greater risk of criminal exploitation, and exploitation tactics used by OCGs. Following a systematic search procedure, five studies were included, most of which were literature reviews. Only one of the included studies provided information on the prevalence rates of criminal exploitation in university students, specifically related to County Lines involvement (Hall et al., 2022), although one explored the likelihood that students might engage in County Lines scenarios following exposure to a number of hypothetical exploitation scenarios (Burt et al., 2022). Prevalence rates from these studies are difficult to interpret, however. For example, Hall et al.’s (2022) reporting of 42 to 109 university students having some involvement in County Lines does not provide sufficient data to allow meaningful conclusions to be drawn regarding prevalence, given that there is no meaningful benchmark to assess prevalence against. Without this data, it is not possible to ascertain what percentage of students from the seven universities that provided data, were victims of criminal exploitation. The methodology employed in Burt et al.’s (2022) study also impacts on the ability to meaningfully interpret prevalence; particularly given they assessed likelihood to engage in County Lines scenarios as opposed to actual engagement.

Nevertheless, more meaningful conclusions can be drawn regarding risk factors for criminal exploitation. In the current review, several commonly reported characteristics associated with criminal exploitation risk were found, with three studies highlighting financial instability and problematic use of substances, respectively, and two mentioning social media usage, living in student accommodation, and students’ inability to decode criminal intentions particularly regarding fake job advertisements, as key risk factors. Many of these findings are consistent with literature that outlines criminal exploitation risk in non-university samples (Hayman et al., 2024; National Crime Agency, 2017; Warburton, 2024) and highlights a significant problem: these risk factors are already highly prevalent in university student populations (Kircaburun et al., 2020 Office for National Statistics, 2023; Students Organising for Sustainability, 2023)

Contrary to expectations, however, Burt et al. (2022) found positive correlations between engagement and mental health well-being, and negative correlations between financial distress and engagement. These findings are explained by the small amount of variance demonstrated by their analysis and the likely presence of other, unobserved factors which may have accounted for their participant responses. Furthermore, whilst Burt et al. (2022) also reported potential demographic risk factors such as age, gender, ethnicity, and whether students were home students or international, given there was no demographic-matched comparison group it is difficult to make conclusions on how these relate to exploitation risk. This is compounded by the use of <50% and >50% engagement scores as a measure of exploitation risk, which may lack reliability and validity. It is important to be cautious when interpreting the results of this study, given it focused on hypothetical vignettes and therefore it is not possible to conclude that participants responses were true indictors of actual behavior.

Findings from this review also highlighted commonly used tactics for criminal exploitation by perpetrators. Notable tactics included debt bondage, fake jobs and get money quick schemes, advertising through social media channels, and cuckooing accommodation. These findings are also consistent with literature that has explored exploitation tactics used in non-student populations (Barclays, 2023; Stone, 2018), and again presents an area of concern given the prevalence of characteristics of university students and that are associated with a greater risk of these means of exploitation.

### Strengths and Limitations

The strength of the current review is that it is the first synthesis of evidence pertaining to criminal exploitation in university students. Police practitioners working to address organized crime know that this subset of the general population are likely to be targeted (Brewster et al., 2022) and yet, to date, there is a paucity of research exploring the vulnerability of university students compared to other victim groups such as children or people with learning difficulties and challenges (Windle et al., 2020). Protecting university students from OCGs can only happen once more is known about what goes on and why. A further strength of the review is that it collates evidence regarding criminal exploitation of university students from a range of countries, highlighting that this is a widespread concern warranting international attention.

In terms of limitations, it is interesting that only a small number of papers arose from the rapid review. Five published works were included, and only one of which was an empirical study. Furthermore, the included studies demonstrated a range of methodological limitations which impacted on their quality. Our study relied on collating evidence from a number of literature reviews and one empirical study published in the UK. Given the methodological differences inherent in these types of sources, it is difficult to explore comparisons and make meaningful conclusions from these studies, particularly regarding prevalence. The fact that our review only included one empirical study which was from the UK also presents a limitation in that we are not able to confidently generalize the findings of this study across the different countries included in our review. A further limitation was that none of the included studies adopted a qualitative research design which would have provided some in-depth insight into how university students manage the vulnerabilities associated with criminal exploitation from drug OCGs.

### Future Directions

#### Implications for Higher Education Institutions (HEI’s)

Tackling drugs and associated sequelae is part of the remit of the Association of University Chief Security Officers (2025) and it is clear that UK universities are already aware that their campuses and accommodation sites offer OCGS opportunities both to sell drugs and/or to recruit into the operational side of the drug running business. The Home Office commissioned Violence and Vulnerability Unit (VVU) have conducted a series of locality reviews in HEI’s in the UK (e.g., London Higher, 2023) and have provided recommendations designed to improve awareness of reporting concerns; cross partnership working and improved situational prevention practices. Incident reporting systems which include data capture about drugs are common in UK universities as are different systems for students to report concerns anonymously. However, specific practices vary across HEI’s and peer reviewed research about the effectiveness of different initiatives is limited. This should be addressed as a priority in order to inform an AUCSO-led best practice guidance document in relation to preventing and protecting university students from criminal exploitation in relation to County Lines.

#### Directions for Further Research

The findings within this review suggest areas for further exploration within future research. This should include universities working with the police service to capture prevalence rates of criminal exploitation in university students, whilst also ensuring that this data can be meaningfully compared to non-selected samples. Due to the paucity of research in this area and the increasing financial pressures that university students are under, there is an urgent need for empirical studies to be undertaken and that should include both quantitative and qualitative research. In terms of the latter, there is currently a lack of qualitative research that has been conducted in this area, with no published works exploring the experiences of students that have been criminally exploited, through the use of data-rich collection methods. Obtaining insights on the lived experience of university students in the context of criminal exploitation is important to fully understand the mechanisms which underpin risk, as well as the negative impact that criminal exploitation has on this population. Whilst there are inherent difficulties in conducting research in this area, these difficulties should not preclude it from happening. Furthermore, future research should look to address the gap in experimental evidence regarding risk factors for criminal exploitation in the university student context, prompted by our included studies. A more established body of rigorous research in this area identifying risk factors would inform future directions for universities across the UK, including a review of policies and procedures and initiatives for closer collaboration with housing, drug support, and policing agencies. Our review included research from several countries, however, given the methodological differences it is difficult to make meaningful comparisons between countries. Future research could focus on highlighting the similarities and differences in criminal exploitation risk for university students according to their geographical location.
